# Antitumor Effect of Water Decoctions of *Taxus* Cuspidate on Pancreatic Cancer

**DOI:** 10.1155/2014/291675

**Published:** 2014-02-25

**Authors:** Chao Qu, Zhen Chen

**Affiliations:** ^1^Department of Integrative Oncology, Fudan University Shanghai Cancer Center, Shanghai 200032, China; ^2^Department of Oncology, Shanghai Medical College, Fudan University, Shanghai 200032, China

## Abstract

The *Taxus* cuspidate has been used as a traditional Chinese medicinal herb and considered to affect various physiological functions in the body for thousands of years. As we know that taxol isolated from the *Taxus* cuspidate has been approved for the treatment of ovarian cancer, it has also shown its antitumor abilities against other kinds of cancers. But the antitumor activity of other components which are free of paclitaxel and hydrophilic paclitaxel derivatives from *Taxus* cuspidate has not been fully understood. In this study, we investigated the effect of the water decoctions from the leaves of *Taxus* cuspidate on pancreatic cancer cell proliferation and the potential mechanism(s); though its antitumor activity and mechanism *in vitro* remain to be elucidated, the water soluble constituents from *Taxus* cuspidate could be used in clinical for cancer patients.

## 1. Introduction

In traditional medicinal history, herbal medicines, which have been used to treat different medical conditions, is now considered a promising choice for treating cancers [1]. Meanwhile, water decoctions from leaves of *Taxus* cuspidate have been used for the treatment of human pancreatic cancers for many years in the cancer hospital, Fudan University, Shanghai, China. Recently, much efforts have been put on discovering pharmacological effects of TCM on tumor cells, and some underlying mechanisms have been revealed, such as inhibition of tumor invasion and induction of cell apoptosis [2].

*Taxus*, a genus of yews, are small coniferous trees or shrubs in the yew family Taxaceae and the natural source of Taxol which is a new antitumor drug, to treat patients with lung, ovarian, breast, head, and neck cancers [3]. Following approval by the US Federal Drug Administration (FDA), paclitaxel (Taxol) has been established as a standard drug for cancer chemotherapy [4, 5], and it has also exhibited its antitumor effect in many clinical and nonclinical trials to treat pancreatic cancer [6, 7]. Inducing a G2/M cell cycle arrest by disrupting disassembly of microtubules is the primary mechanism of Taxol on tumor progression [8].

Following the first observation of the antitumor activities *in vivo* and *in vitro* of the water extract [9], researches have showed that aqueous extract of *Taxus* chinensis could significantly inhibit the proliferation of A549 cells and induce the apoptosis by regulating the expressions of surviving [10]. Polysaccharides contents dissolved in water from *Taxus yunnanensis* displayed mild cytotoxicity against cancer cells in a concentration-dependent manner [11]. Unlike the clear antitumor mechanism of Taxol, the antitumor mechanism of components which are free of paclitaxel from *Taxus* cuspidate is still on the way to be uncovered. Here, the water decoction of *Taxus* cuspidate was prepared, its antiproliferation effect was determined, and the potential mechanisms were explored in human pancreatic cancer.

## 2. Materials and Methods

### 2.1. Preparation of Water Decoctions from *Taxus* Cuspidate

Air-dried leaves of *Taxus* cuspidate were supplied by the Company of Ningbo Taikang yew biological engineering Co, Ltd. The final decoction of the *Taxus* cuspidate was prepared at the Department of Pharmacy, Fudan University Shanghai Cancer Center, Shanghai, China, by boiling them with distilled water to the required concentration, the daily dosage as described previously [12]. Drug administration was started from 1 week after the injection of tumor cells and continued until the end of the experiment. Body weights were recorded once per week. Mice of the treatment groups were orally administered water decoction (5 g/kg, 10 g/kg, and 20 g/kg) with 0.2 mL each time. Mice of the control group were administered normal saline (10 mice in each group). Treatment point was continued for 4 weeks, and then mice were sacrificed and tumors were removed and weighed. Tissues that would be used for molecular biological analysis were preserved in neutral-buffered formalin at 4°C before embedding in paraffin.

### 2.2. Cell Lines and Animals

Human pancreatic cancer cell line capan1 was obtained from the American Type Culture Collection and cultured in DMEM supplemented with 10% FBS in a humidified incubator containing 5% CO_2_ atmosphere at 37°C. Female BALB/c-nu/nu nude mice, 4 to 6 weeks sold, were obtained from the Laboratory Animal Center, Shanghai University of Traditional Chinese Medicine, and housed in laminar flow cabinets under specific pathogen-free conditions and provided with food and water ad libitum.

### 2.3. Mouse Model of Pancreatic Cancer *In Vivo*

40 female BALB/c-nu/nu nude mice weighing 18–20 g were used; briefly, 2 × 10^6^ capan1 pancreatic cancer cells per animal in 200 ul of phosphate-buffered saline were subcutaneously injected into the flank of each mouse. The mice are randomly divided into 4 groups, each experimental group contains 10 mice. Length and width of tumors (in millimeters) were measured twice a week with calipers. Tumor volumes were calculated by the formula (*a* × *b* ^2^) × 0.5, where *a* and *b* are the long and short dimensions, respectively. After four-week observation, the mice were sacrificed, tumors were resected, and tumor weights were measured.

### 2.4. H and E Staining and Immunohistochemistry

For H and E staining, paraffin-embedded sample slides were deparaffinized, hydrated, and then stained with hematoxylin for 1 min. After rinsing, the slides were stained with eosin for 1 min, rinsed, and sealed with cover slips using Permount. Immunohistochemistry (IHC) was performed as described previously [13]. Briefly, specimens of tumor tissue were fixed in 10% formalin and embedded in paraffin wax. Unstained 3 mm sections were then cut from the paraffin blocks for IHC analysis. The sections were stained with rabbit anti-ki67 (1 : 500), rabbit anti-P65 (1 : 250), and rabbit anti-cyclin D1 (1 : 100) at 4°C overnight. The procedures were performed by two independent investigators and one pathologist, all of whom were blinded to the model/treatment type for the series of specimens. The determination of Ki67 positive cells was performed according to the protocol of the Ki67 immunohistochemistry kit. The average rate of Ki67 positive cells in one tumor sample was calculated.

### 2.5. Cell Cycle Analysis

3 transplanted tumor samples were selected randomly from each group, each weights 500 mg. The tumor samples were washed with PBS in 4°C and cut into pieces with a blade. The residual pieces were grinded into single-cell suspension through a 200 hole mesh screen, the single-cell suspension was centrifuged in 1000 r/min for 5 minutes, and modulated into 10^6^/mL. Cells were fixed with 1% paraformaldehyde in PBS (phosphate-buffered saline) for 15 minutes and refixed with 70% ethanol. The cells were then treated following the standardized protocol, and cell cycle analyses were done by flow cytometry.

### 2.6. RNA Isolation and qRT-PCR

Total RNA was isolated from the transplanted tumor resection using Trizol Reagent (Invitrogen, San Diego, CA, USA) and reverse transcription PCR (RT-PCR) was performed according to the manufacturer's instructions (MBI Fermentas, Vilnius, Lithuania). Quantitative real-time PCR was performed in 96-well plates using SYBR Green MasterMix (Applied Biosystems) on an iCycler system (Bio-Rad). The following PCR primer pairs were used: IL-1*β*, 5′-GGCAATGAGGATGACTTGTTCT-3′ (forward) and 5′-CTGTAGTGGTGGTCGGAGATTC-3′ (reverse); IL-6, 5′-TCATCACTGGTCTTTTGGAGTT-3′ (forward) and 5′-GCTCTGGCTTGTTCCTCACTAC-3′ (reverse); IL-8, 5′-CTGGACCCCAAGGAAAACTG-3′ (forward) and 5′-CCCTACAACAGACCCACACAAT-3′ (reverse) and GAPDH, 5-gctgtgtggcaaagtccaag-3 (forward) and 5-ggtcaggctcctggaagata-3 (reverse). The thermal conditions for real-time PCR assays were as follows: cycle 1, 95°C for 10 min; cycle 2, (×40), 95°C for 10 s and 58°C for 45 s.

### 2.7. Western Blot Analysis

Western blot analysis was performed as described in our previous report [13]. Briefly, proteins were extracted from transplanted tumor resection and quantified with the bicinchoninic acid (BCA) assay kit (Pierce, Rockford, IL, USA) using BSA as a standard and assay kit (Pierce, Rockford, IL, USA) using BSA as a standard. Equal amounts of protein (50 mg) were separated using 10% SDS-PAGE and transferred to PVDF membranes (MILLIPORE). The membranes were blocked with 5% nonfat milk and incubated with primary antibodies. The target proteins were detected using an enhanced chemiluminescence (ECL) kit (Amersham Pharmacia Biotech, Uppsala, Sweden).

### 2.8. Statistical Analysis

Statistical analysis was performed using SPSS 16.0 software. The results are expressed as mean ± SD. Dunnett's test was used to evaluate the significance of differences between the treated and control groups. Differences of parameters were analyzed by one-way ANOVA. A value of *P* < 0.05 was considered as statistically significant.

## 3. Results

### 3.1. Inhibitory Effect of Water Decoction from *Taxus* Cuspidate on Pancreatic Tumor Growth

**Figure 1:**
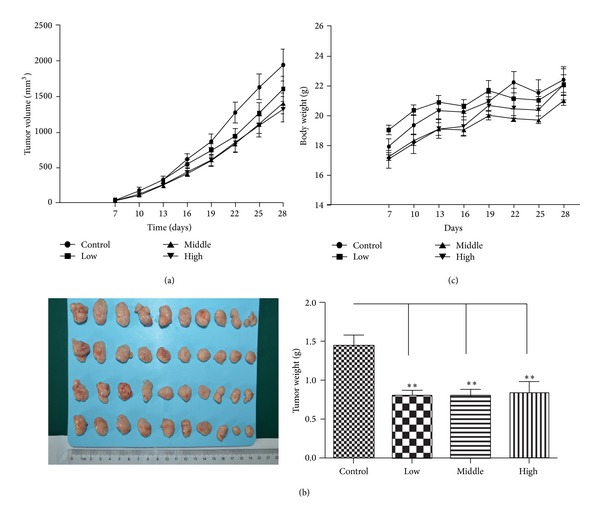
Inhibitory effect of water decoction from *Taxus* cuspidate on pancreatic tumor growth *in vivo*. capan1 cells (2 × 10^6^ cells in 200 mL) were injected subcutaneously into the right axilla of each BALB/c-nu/nu nude mouse. One week later, mice were orally treated with or without water decoction from *Taxus* cuspidate. Mean tumor volumes of control (saline water) and treated tumors were measured. Mean ± standard deviation was determined for 10 mice in each treatment group. The tumor growth curves are shown in (a). The tumor samples of each group are shown in (b); the body weight curves are shown in (c).

To explore the role of water decoction from *Taxus* cuspidate on pancreatic cancer growth *in vivo*, nude mice which were injected with pancreatic cancer cells were divided randomly into four groups: one group received normal saline as a control and the other received the water decoction. The tumor-bearing mice were treated with low (5 g/kg), middle (10 g/kg), and high (20 g/kg) concentrations, and water decoction was administrated from 1 week after the injection of tumor cells and continued until the end of the experiment. The treatment was administrated for 28 days. All of the experimental mice survived the whole management, on the 28th day, the data of tumor volume and tumor weight was collected, according to the tumor volumes, the tumor growth of the treatment groups were inhibited. The tumor weight of the treatment groups were lower than that of the control group (*P* = 0.0006; *P* = 0.0009; *P* = 0.0076), respectively (Figure 1). The tumor growth inhibition ratio of the treatment groups were 45.08%; 44.93% and 43.16%, respectively (Table 1). Moreover, no significantly differences were shown among the treatment groups. In addition, no differences of body weight were observed between the treatment groups and control group (Figure 1). As the results show *in vitro*, we do the subsequently experiments to uncover the antitumor mechanism.

### 3.2. Water Decoction of *Taxus* Cuspidate Induces a G1 Cell Cycle Arrest on Tumor Growth

**Figure 2:**
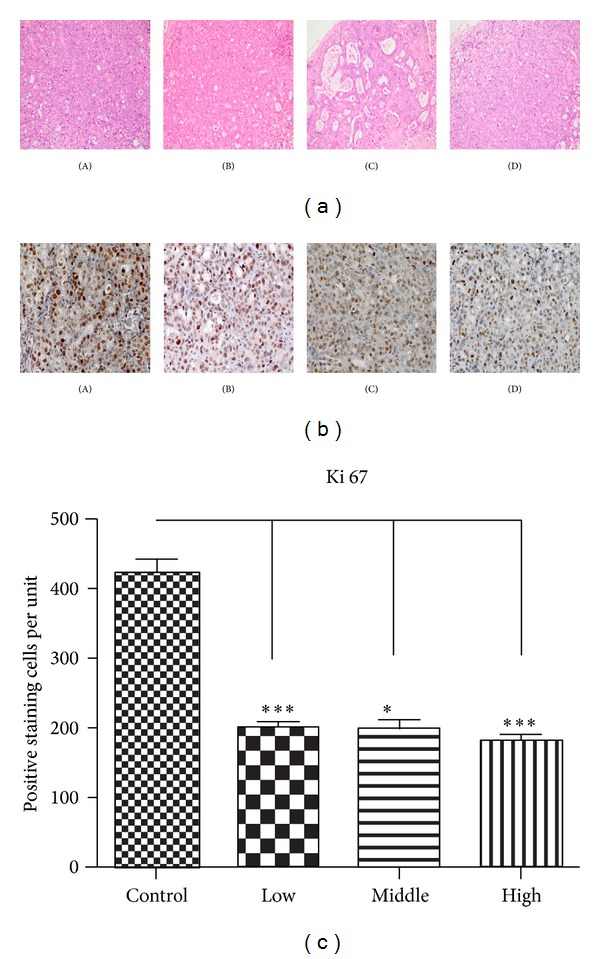
Examination of marker of cell proliferation ki67. (a) H and E staining was performed using sections of transplanted tumors from each group. Original magnification ×200. (b) Expression of Ki67 which is the marker of cell proliferation was performed by immunohistochemical examination in tumor tissues isolated from control and the treatment groups. Original magnification ×400. (c) Ki-67 positive staining cells were evaluated quantitatively by calculating the number of cells per unit, and the mean value from ten fields under 400x microscopy was determined. ***P < 0.001. (A) Control group, (B) low concentration group, (C) middle concentration group, and (D) high concentration group.

**Figure 3:**
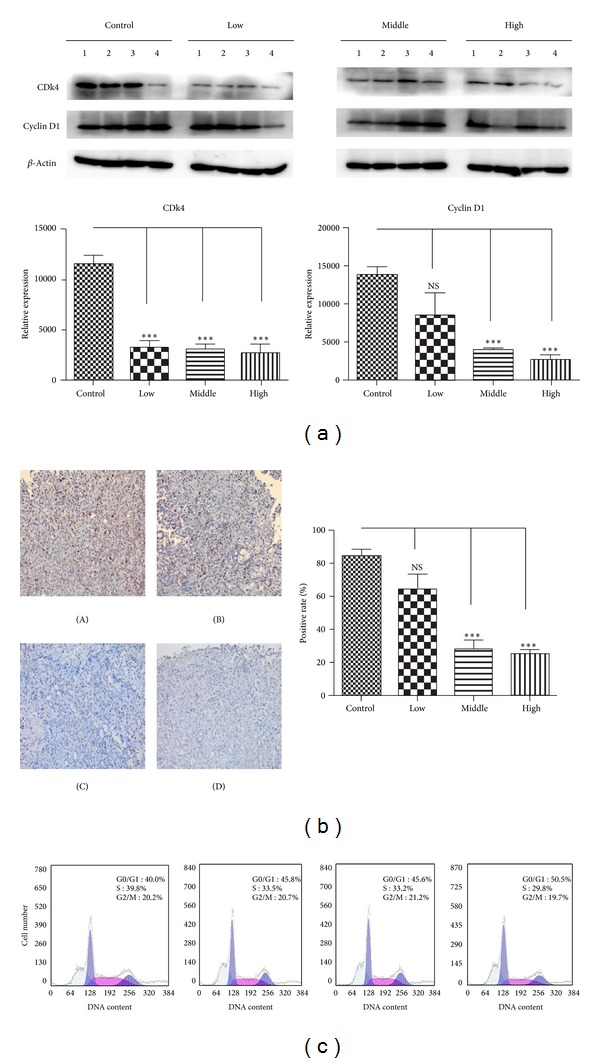
Water decoction of *Taxus* cuspidate induces a G1 cell cycle arrest on tumor growth. (a) Western blot analysis was used to measure the expression of cyclin D1 and CDK4 in cells derived from four representative implanted samples from each group. The band intensities were measured by densitometry and the relative indicated protein expression was shown. **P < 0.01 compared with the control group. (b) IHC staining with anti-cyclin D1 antibody was performed using sections of transplanted tumors from each group. Cyclin D1 was evaluated quantitatively by calculating the ratio of the cyclin D1-positive area to the total area in each field and the mean value from 5 fields under 200x microscopy was determined. Original magnification ×200. (c) Single-cell suspension prepared from transplanted tumor resection was analyzed by flow cytometry to determine the number of cells in G0/G1, S, and G2/M phases. (A) Control group, (B) low concentration group, (C) middle concentration group, and (D) high concentration group.

As the results showed above, we supposed that the water decoction may inhibit the tumor through disrupting the proliferation of tumor cells, so we stained the proliferation related antigen, Ki-67, which is overexpressed by proliferative tumor cells. The expression of the Ki-67 protein is strictly associated with proliferation of tumor cells, and the antigen can be exclusively detected within the nucleus, whereas in mitosis most of the protein is relocated to the surface of the chromosomes [14]. The Ki-67 protein is present during all active phases of the cell cycle but is absent from resting cells [15] in this study, the number of cells expressing the proliferation marker Ki-67 was found to be reduced in sections of treatment groups compared with the control group (Figure 2). So we evaluated the cyclin and CDK expressions which control the cancer cell cycle aberrant activation of cyclin D1-Cdk4 signaling pathway is commonly found in pancreatic ductal adenocarcinoma, the increased expression of cyclin D1 and CDK4 leads to the activation of cyclin D1-Cdk4 signaling and unchecked proliferation, and cyclin D1 is also the target gene of Wnt/*β*-catenin cell signaling which is known to promote the neoplastic transformation in PDA [16]. Immunohistochemisty and/or western blot analysis revealed that the treatment of the water decoction suppressed the expression of cyclin D1 and CDK4 (Figure 3). Flow cytometric analyses revealed that the percentage of treatment group cells in the G1 phase dramatically increased compared with the control group (Figure 3). These data suggest that the water decoction may inhibit growth of pancreatic cancer cells by suppressing the cyclin D1 and CDK4, resulting in the G1 cell cycle arrest.

### 3.3. The Water Decoction of *Taxus* Cuspidate Prevents the Pancreatic Cancer Cell Proliferation by Disrupting the wnt/*β*-Catenin Cell Signaling

**Figure 4:**
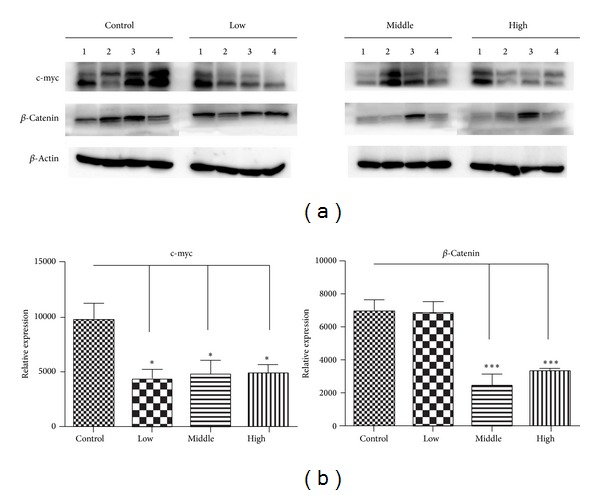
The water decoction of *Taxus* cuspidate prevents the wnt/*β*-catenin cell signaling. Western blot analysis was used to measure the expression of c-myc and *β*-catenin in cells derived from four representative implanted samples from each group. The band intensities were measured by densitometry and the relative indicated protein expression was shown. *P < 0.05; **P < 0.01; ***P < 0.001 compared with the control group.

wnt ligands expression and activation of the wnt/*β*-catenin pathway have been associated with pancreatic ductal adenocarcinoma [17], and a growing number of agents targeting ligand-induced wnt/*β*-catenin signaling are being developed for cancer therapy [18]. Cyclin D1 and c-myc are two classic target genes of wnt/*β*-catenin signaling which are related to proliferation [19]. As the results show above, the expression of cyclin D1 is significantly inhibited, and we assumed that the water decoction of *Taxus* cuspidate may disrupt the wnt/*β*-catenin cell signaling to inhibit the proliferation of pancreatic cancer. We tested another target gene of wnt/*β*-catenin signaling. We found that the expression of c-myc is also reduced. Conventional wnt signaling causes *β*-catenin accumulation in a complex with the transcription factor TCF/LEF that regulates target gene expression in the absence of wnt signaling, the level of *β*-catenin is kept low through degradation by GSK-3*β* and located in the E-cadherins at the plasma membrane. When the wnt ligands bind to its receptors, *β*-catenin escapes the degradation fate and translocates into the cell nucleus [20]. So we tested the *β*-catenin expression by western blot analysis, and we found that the protein expression of *β*-catenin is also downregulated (Figure 4). In summary, the water decoction of *Taxus* cuspidate prevents the pancreatic cancer cell proliferation by disrupting the wnt/*β*-catenin cell signaling.

### 3.4. The Water Decoction of *Taxus* Cuspidate Suppresses Cancer-Related Inflammation in Pancreatic Cancer

**Figure 5:**
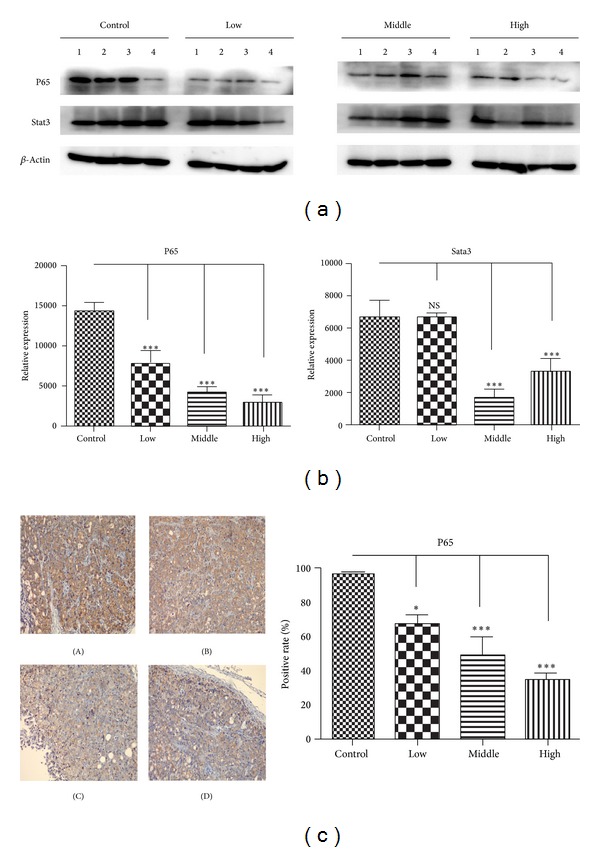
The water decoction of *Taxus* cuspidate suppresses cancer-related inflammation in pancreatic cancer. (a) Western blot analysis was used to measure the expression of inflammatory regulator NF-KB (P65) and stat3 in cells derived from four representative implanted samples from each group. The band intensities were measured by densitometry and the relative indicated protein expression was shown. ***P < 0.001 compared with the control group. (b) IHC staining with anti-p65 antibody was performed using sections of transplanted tumors from each group. P65 was evaluated quantitatively by calculating the ratio of the P65-positive area to the total area in each field and the mean value from 5 fields under 200x microscopy was determined. Original magnification ×200. (A) Control group, (B) low concentration group, (C) middle concentration group, and (D) high concentration group.

**Figure 6:**
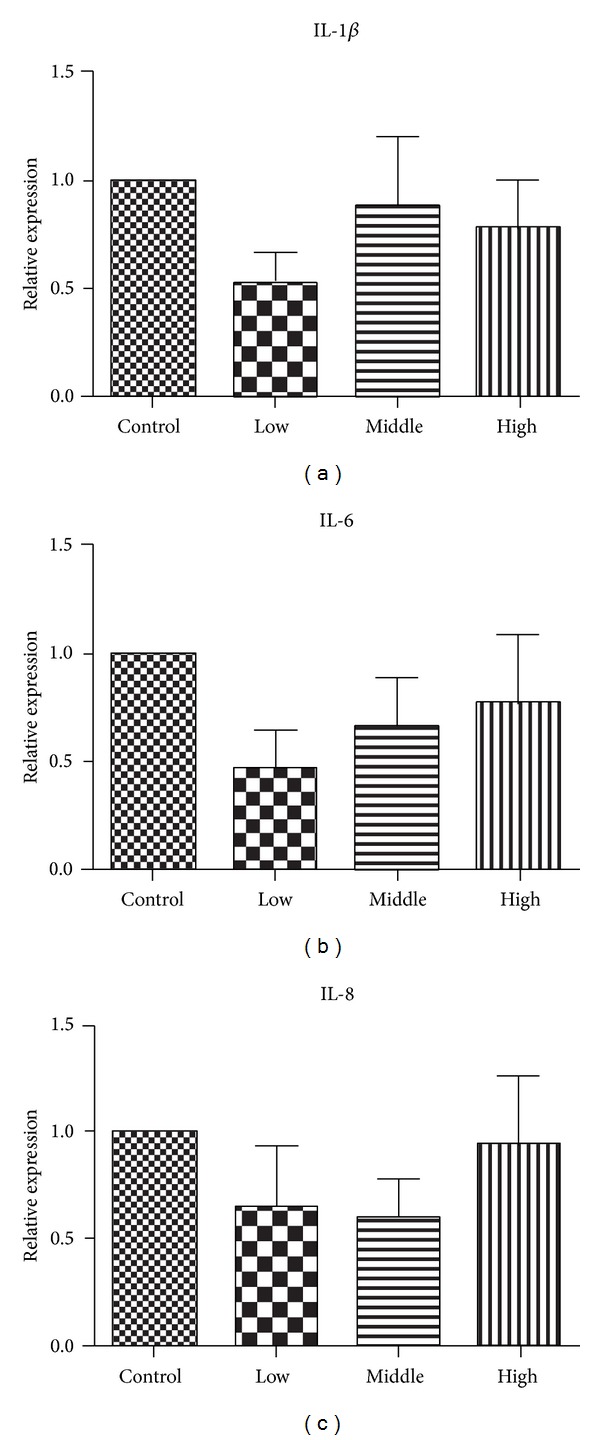
Effect of water decoction from *Taxus* cuspidate on inflammatory cytokines expression; IL-1*β*, IL-6, and IL-8. IL-1*β*, IL-6, and IL-8 mRNAs of tumors of each group were detected by reverse transcription polymerase chain reaction (RT-PCR), the relative expression of each cytokines is shown.

Pancreas acinar cell can undergo ductal metaplasia in the inflammatory environment of pancreatitis. This metaplastic change is now recognized as a precursor of pancreatic cancer. Inflammatory cytokines which are secreted by the epithelium and the stromal also promote tumor growth through autocrine and paracrine effects [21]. Numerous studies have indicated that cancer-related inflammation promotes the development of tumors [22, 23], and NF-KB (P65) and stat3 are two core factors involved in the inflammatory cytokines stimulating pancreatic tumor proliferation [24–26]. Therefore, we evaluated the NF-KB (P65) and stat3 expressions by immunohistochemisty and/or western blot analysis, and our data demonstrated that the water decoction of *Taxus* cuspidate suppressed the expression of them compared with the control group (Figure 5); as we know, IL-1*β*, IL-6, and IL-8 are classic inflammatory cytokines that stimulate the activation of NF-KB or stat3 cell signaling [25, 26]. But the qRT-PCR analysis shows no differences between the control and treatment groups (Figure 6). Taken together, all these data clearly demonstrated that the water decoction could suppress the inflammation related cell signaling without affecting the stimulating factors, such as IL-1*β*, IL-6, and IL-8.

## 4. Discussion

Pancreatic cancer is the fourth leading cause of cancer-related death in US, with a potential 3- to 6-month survival benefit and an overall 5-year survival rate of less than 5% [27]. A majority of patients are presented with locally or distant metastasis for the first time of diagnosis.

With the characteristic of insensitivity to chemotherapy and radiotherapy, searching more effective methods to treat pancreatic cancer is urgent. Traditional Chinese medicine (TCM) has been widely used for cancer treatment in China for thousands of years. Application of integrative TCM and Western medicine to treat pancreatic cancer has shown promising results. Our previous studies have shown that TCM could inhibit the tumor growth *in vivo* and *in vitro* by interfering in many mechanisms [28, 29]. As the rare resources of Taxol which is approved for the treatment of kinds of tumors, the conventional application of *Taxus* cuspidate, different from the Western medicines, is also used in cancer patients' treatment. Some studies have already uncovered some antitumor mechanisms of *Taxus* cuspidate in cancers, but much more efforts are still needed, especially in pancreatic cancer.

Herbal medicines usually are evaluated as alternative treatments for cancer. The traditional way to prepare the herb is to boil it in water. So we take insight into it to find out the mechanism of antitumor mechanism. In the present study, we investigated tumor inhibition effect of the water decoction of *Taxus* cuspidate, and the tumor weight and tumor volumes are significantly decreased. The immunohistochemisty analysis showed that the extent and intensity of Ki-67 expression are also reduced. The expression of the Ki-67 protein is strictly associated with proliferation of tumor cell, and recent studies have demonstrated that deregulation of cell cycle control may contribute to the unlimited cancer cell proliferation. Cyclins and CDKs are two positive regulators which control the growth of cancer cells, and aberrant activation of cyclin D-Cdk4 signaling pathway is commonly found in pancreatic ductal adenocarcinoma (PDA). Cyclin D1 is also the downstream target gene of wnt/*β*-catenin cell signaling which mediates the progression of PDA [16]. Moreover, cyclin D1 overexpression in PDAC correlates with poor postoperative patient survival [30]. Therefore, it is possible that the water decoction of *Taxus* cuspidate which may inhibit expression of these cyclin D1 and CDK4 to suppress the tumor growth. In this study, we showed that the water decoction of *Taxus* cuspidate significantly inhibited the expressions of two regulators.

Embryonic signaling pathways control proper organ formation during development. Increasing evidence suggests that these pathways remain active in a subpopulation of cells within adult organs and that deregulation of their activity contributes to the development and progression of tumors [31]. Notch, Sonic Hedgehog, and wnt/*β*-catenin signaling pathways are involved in the progression of pancreatic cancer [16, 32, 33]. Cyclin D1 and c-myc are two classic target genes of wnt/*β*-catenin signaling which are related to proliferation [19], cyclin D1 overexpression in PDAC correlates with poor postoperative patient survival [30], and c-myc protein expression is connected to the transcription of the E2F1 gene in PDAC cells, leading to S-phase progression of the cell cycle [34]. So in our study, we tried to evaluate the expression of c-myc as the result shows, c-myc is decreased after the treatment of the water decoction of *Taxus* cuspidate. Therefore, we hypothesized that it may disrupt the wnt/*β*-catenin signaling to inhibit the progression of pancreatic cancer. Canonical wnt/*β*-catenin signaling is activated when soluble wnt ligands bind to one of several FRIZZLED receptors and LRP5/LRP6 coreceptors, and this interaction results in the inhibition of *β*-catenin phosphorylation, so *β*-catenin translocates from the cytoplasm to the nucleus where it binds to the TCF-LEF family of transcription factors to activate the transcription of wnt target genes: cyclin D1 and c-myc. *β*-catenin which is the core factor is located in the E-cadherins at the plasma membrane when wnt signaling is inactivated. So we investigated the expression of *β*-catenin. Our results show that the protein expression of it is decreased, which indicated that the water decoction of *Taxus* cuspidate disrupts the wnt/*β*-catenin cell signaling.

Recent studies have confirmed that inflammatory environment could affect many aspects of malignancy, including the proliferation, angiogenesis, metastasis, and response to treatment [23]. In pancreatic cancer, chronic pancreatitis predisposes to pancreatic cancer development and both diseases share a common etiology [21]. The pancreatic tumor microenvironment is full of growth factors and inflammatory cytokines that support tumor growth, and it is highly immunosuppressive [35]. Apart from the secreted cytokines in the microenvironment, kinds of inflammatory cells of host also contribute to the progression of pancreatic cancer, such as tumor-associated macrophages, cancer-associated stellate cells, and educated fibroblast of the stroma [36–38]. NF-KB (P65) and stat3 are two pivotal factors, activated by inflammatory cytokines, such as IL-1*β*, IL-6, and IL-8, which contribute to the proliferation of pancreatic cancer [39–42]. According to our results, the water decoction of *Taxus* cuspidate attenuates the inflammatory activation by downregulating the two important factors, but without affecting the stimulating inflammatory cytokines. In conclusion, traditional Chinese medicine could be used alone, or as an adjuvant in combination with chemotherapeutic drugs for cancer treatment, including *Taxus* cuspidate. There are many difficulties in studying the molecular mechanisms of water decoction of TCM which are totally different from the extract agent. Though the underlying antitumor mechanisms of water decoction from TCM are still unclear, future studies *in vitro* will be helpful to uncover it.

## Figures and Tables

**Table 1:** Inhibition of pancreatic cancer transplanted in mice.

Groups	Animal number start/end	Tumor weight (g) X ± SD	Inhibition rate (%)
Control	10/10	1.45 ± 0.40	
Low concentration (5 g/kg)	10/10	0.79 ± 0.22	45.08%
Middle concentration (10 g/kg)	10/10	0.80 ± 0.26	44.93%
High concentration (20 g/kg)	10/10	0.82 ± 0.39	43.16%

(*P* < 0.01 compared with the control group).

## References

[1] Xu Z, Chen X, Zhong Z, Chen L, Wang Y (2011). Ganoderma lucidum polysaccharides: immunomodulation and potential anti-tumor activities. *The American Journal of Chinese Medicine*.

[2] Li-Weber M (2013). Targeting apoptosis pathways in cancer by Chinese medicine. *Cancer Letters*.

[3] Skwarczynski M, Hayashi Y, Kiso Y (2006). Paclitaxel prodrugs: toward smarter delivery of anticancer agents. *Journal of Medicinal Chemistry*.

[4] Lin YJ, Zhen YZ, Shang BY, Zhen YS (2009). Rhein lysinate suppresses the growth of tumor cells and increases the anti-tumor activity of taxol in mice. *The American Journal of Chinese Medicine*.

[5] Koolen SL, Beijnen JH, Schellens JH (2010). Intravenous-to-oral switch in anticancer chemotherapy: a focus on docetaxel and paclitaxel. *Clinical Pharmacology and Therapeutics*.

[6] von Hoff DD, Ervin T, Arena FP (2013). Increased survival in pancreatic cancer with nab-paclitaxel plus gemcitabine. *The New England Journal of Medicine*.

[7] Jeansonne DP, Koh GY, Zhang F (2011). Paclitaxel-induced apoptosis is blocked by camptothecin in human breast and pancreatic cancer cells. *Oncology Reports*.

[8] Andreopoulou E, Muggia F (2008). Pharmacodynamics of tubulin and tubulin-binding agents: extending their potential beyond taxanes. *Clinical Breast Cancer*.

[9] Jiang S, Zhang Y, Zu Y, Wang Z, Fu Y (2010). Antitumor activities of extracts and compounds from water decoctions of Taxus cuspidata. *The American Journal of Chinese Medicine*.

[10] Shu QJ, Li P, Wang BB (2011). Experimental study on apoptosis induced by aqueous extract of Taxus chinensis in human pulmonary carcinoma cell A549 and its molecular mechanisms. *Zhongguo Zhong Xi Yi Jie He Za Zhi*.

[11] Yan C, Yin Y, Zhang D, Yang W, Yu R (2013). Structural characterization and in vitro antitumor activity of a novel polysaccharide from Taxus yunnanensis. *Carbohydrate Polymers*.

[12] Chen Z, Chen LY, Wang P, Dai HY, Gao S, Wang K (2012). Tumor microenvironment varies under different TCM ZHENG models and correlates with treatment response to herbal medicine. *Evidence-Based Complementary and Alternative Medicine*.

[13] Wang P, Chen Z, Meng ZQ (2009). Dual role of Ski in pancreatic cancer cells: tumor-promoting versus metastasis-suppressive function. *Carcinogenesis*.

[14] Yang JX, Fichtner I, Becker M, Lemm M, Wang XM (2009). Anti-proliferative efficacy of icariin on HepG2 hepatoma and its possible mechanism of action. *The American Journal of Chinese Medicine*.

[15] Scholzen T, Gerdes J (2000). The Ki-67 protein: from the known and the unknown. *Journal of Cellular Physiology*.

[16] Pasca di Magliano M, Biankin AV, Heiser PW (2007). Common activation of canonical Wnt signaling in pancreatic adenocarcinoma. *PLoS ONE*.

[17] Zhang Y, Morris JPT, Yan W (2013). Canonical Wnt signaling is required for pancreatic carcinogenesis. *Cancer Research*.

[18] Jiang X, Hao HX, Growney JD (2013). Inactivating mutations of RNF43 confer Wnt dependency in pancreatic ductal adenocarcinoma. *Proceedings of the National Academy of Sciences of the United States of America*.

[19] Klaus A, Birchmeier W (2008). Wnt signalling and its impact on development and cancer. *Nature Reviews Cancer*.

[20] Mosimann C, Hausmann G, Basler K (2009). *β*-catenin hits chromatin: regulation of Wnt target gene activation. *Nature Reviews Molecular Cell Biology*.

[21] Pinho AV, Chantrill L, Rooman I (2013). Chronic pancreatitis: a path to pancreatic cancer. *Cancer Letters*.

[22] Balkwill F, Charles KA, Mantovani A (2005). Smoldering and polarized inflammation in the initiation and promotion of malignant disease. *Cancer Cell*.

[23] Mantovani A, Allavena P, Sica A, Balkwill F (2008). Cancer-related inflammation. *Nature*.

[24] Hussain F, Wang J, Ahmed R (2010). The expression of IL-8 and IL-8 receptors in pancreatic adenocarcinomas and pancreatic neuroendocrine tumours. *Cytokine*.

[25] Block KM, Hanke NT, Maine EA, Baker AF (2012). IL-6 stimulates STAT3 and Pim-1 kinase in pancreatic cancer cell lines. *Pancreas*.

[26] Okitsu K, Kanda T, Imazeki F (2010). Involvement of interleukin-6 and androgen receptor signaling in pancreatic cancer. *Genes and Cancer*.

[27] Liu F, Korc M (2012). Cdk4/6 inhibition induces epithelial-mesenchymal transition and enhances invasiveness in pancreatic cancer cells. *Molecular Cancer Therapeutics*.

[28] Wang P, Liu LM, Chen Z (2010). Effect of Qingyi Huaji formula for inhibition of pancreatic cancer cell growth through down-regulating Ski expression. *Zhongguo Zhong Xi Yi Jie He Za Zhi*.

[29] Wang P, Chen Z, Meng ZQ (2010). Ski acts as therapeutic target of Qingyihuaji formula in the treatment of SW1990 pancreatic cancer. *Integrative Cancer Therapies*.

[30] Kornmann M, Ishiwata T, Itakura J, Tangvoranuntakul P, Beger HG, Korc M (1998). Increased cyclin D1 in human pancreatic cancer is associated with decreased postoperative survival. *Oncology*.

[31] Pasca di Magliano M, Hebrok M (2003). Hedgehog signalling in cancer formation and maintenance. *Nature Reviews Cancer*.

[32] Thayer SP, di Magliano MP, Heiser PW (2003). Hedgehog is an early and late mediator of pancreatic cancer tumorigenesis. *Nature*.

[33] Ma J, Xia J, Miele L, Sarkar FH, Wang Z (2013). Notch signaling pathway in pancreatic cancer progression. *Pancreatic Disorders and Therapy*.

[34] Schild C, Wirth M, Reichert M, Schmid RM, Saur D, Schneider G (2009). PI3K signaling maintains c-myc expression to regulate transcription of E2F1 in pancreatic cancer cells. *Molecular Carcinogenesis*.

[35] Roshani R, McCarthy F, Hagemann T (2013). Inflammatory cytokines in human pancreatic cancer. *Cancer Letters*.

[36] Liu CY, Xu JY, Shi XY (2013). M2-polarized tumor-associated macrophages promoted epithelial-mesenchymal transition in pancreatic cancer cells, partially through TLR4/IL-10 signaling pathway. *Laboratory Investigation*.

[37] Mace TA, Ameen Z, Collins A (2013). Pancreatic cancer-associated stellate cells promote differentiation of myeloid-derived suppressor cells in a STAT3-dependent manner. *Cancer Research*.

[38] Seton-Rogers S (2011). Pancreatic cancer: fibroblast co-conspirators. *Nature Reviews Cancer*.

[39] Simone RE, Russo M, Catalano A (2011). Lycopene inhibits NF-KB-Mediated IL-8 expression and changes redox and PPAR*γ* signalling in cigarette smoke-stimulated macrophages. *PLoS ONE*.

[40] Kang R, Tang D, Lotze MT, Zeh HJ (2012). AGER/RAGE-mediated autophagy promotes pancreatic tumorigenesis and bioenergetics through the IL6-pSTAT3 pathway. *Autophagy*.

[41] Zhao G, Zhang JG, Shi Y (2013). MiR-130b is a prognostic marker and inhibits cell proliferation and invasion in pancreatic cancer through targeting STAT3. *PLoS ONE*.

[42] Pugazhenthi S, Zhang Y, Bouchard R, Mahaffey G (2013). Induction of an inflammatory loop by interleukin-1beta and tumor necrosis factor-alpha involves NF-kB and STAT-1 in differentiated human neuroprogenitor cells. *PLoS ONE*.

